# circDNMT1 Promotes Malignant Progression of Gastric Cancer Through Targeting miR-576-3p/Hypoxia Inducible Factor-1 Alpha Axis

**DOI:** 10.3389/fonc.2022.817192

**Published:** 2022-05-30

**Authors:** Hanghang Li, Bo Cao, Ruiyang Zhao, Tian Li, Xingming Xu, Hao Cui, Huan Deng, Jingwang Gao, Bo Wei

**Affiliations:** ^1^ Graduate School, Medical School of Chinese People’s Liberation Army (PLA), Beijing, China; ^2^ First Medical Center, Chinese People’s Liberation Army (PLA) General Hospital, Beijing, China; ^3^ School of Basic Medicine, Fourth Military Medical University, Xi’an, China

**Keywords:** gastric cancer, circDNMT1, miR-576-3p, HIF-1α, cancer progression, glycolysis

## Abstract

**Background:**

Circular RNAs (circRNAs) regulate multiple malignant behaviors of various types of cancer. The role of circDNMT1, a newly identified circRNA, remains unknown in gastric cancer (GC). This study aimed to elucidate the underlying mechanisms of circDNMT1 in regulating GC progression.

**Methods:**

microRNA (miRNA) and circRNA expression was detected by quantitative real-time PCR. Western blotting was performed to measure hypoxia inducible factor-1 alpha (HIF-1α) protein expression. Sanger sequencing, gel electrophoresis and fluorescence *in situ* hybridization were performed to identify the presence of circDNMT1. The clinicopathological features and overall survival of patients were analyzed based on circDNMT1 expression. The proliferation, migration and invasion of GC cells were determined by cell counting kit-8, 5-ethynyl-2’-deoxyuridine, wound healing and transwell assays. Glycolysis of GC cells was detected based on the levels of glucose uptake, the lactate acid, ATP and pyruvic acid production and the extracellular acidification and oxygen consumption rates. The binding sites between miR-576-3p and circDNMT1 or HIF-1α were predicted by online bioinformatic tools and were validated using RNA pull-down and luciferase reporter assays. Xenograft models were established to determine the effects of the circDNMT1/miR-576-3p/HIF-1α axis on GC growth and metastasis *in vivo*.

**Results:**

circDNMT1 was successfully identified and shown to be overexpressed in GC tissues and cell lines. The expression levels of circDNMT1 were correlated with pathological T stage, pathological TNM stage and shorter survival time of GC patients. circDNMT1 knockdown inhibited the proliferation, migration, invasion and glycolysis of GC cells. circDNMT1 functioned as an oncogenic factor by sponging miR-576-3p. HIF-1α was negatively regulated by miR-576-3p *via* binding its mRNA 3’ untranslated region. circDNMT1 promoted malignant behaviors and metabolic reprogramming of GC by targeting the miR-576-3p/HIF-1α axis both *in vitro* and *in vivo*.

**Conclusion:**

These findings demonstrated that circDNMT1 knockdown inhibited GC proliferation, migration, invasion and glycolysis through sponging miR-576-3p/HIF-1α axis. circDNMT1 may be a novel target for GC treatment.

## Introduction

According to the latest epidemiological investigation, gastric cancer (GC) ranks fifth in incidence and forth in mortality worldwide (1). Due to its atypical symptoms, patients are usually diagnosed with GC at advanced or late stages, which limits survival benefits from current therapeutic methods. Gastrectomy and chemotherapy serve as classical regimens, whereas the long-term survival of patients is unsatisfactory. The side effects induced by these interventions also reduce their quality of life. In recent years, targeted drugs have been widely applied as alternative GC treatment regimens (2, 3). Nevertheless, a significant proportion of patients cannot harvest survival benefits from these treatment due mutations in specific gene sites. GC heterogeneity in a single tumor is also an important reason for therapy failure. Cancer cells with drug resistance will be naturally selected out and become the dominant portion of tumors, leading to the insensitivity to targeted drugs. It is urgent to develop regimens that interfere with more common characteristics based on the achievements of basic research.

Circular RNAs (circRNAs) are a type of non-coding RNAs, which are covalently closed and single−stranded (4). circRNAs have a notable stability serves as a feature of circRNAs. Their half−life periods are usually more than 48 h (5). They were first identified in 1976 by Kolakofsky (6) and regarded as the product of error splicing for a long time. With the development of research, circRNAs have been reported to regulate various physiological and pathological processes (7, 8). The biological functions of circRNAs can be primarily classified into four aspects: microRNA (miRNA) sponges, modulation of protein activity, regulation of gene expression and direct translation of peptides (8–11). The most attractive role of circRNA is miRNA sponging, which can abolish the biological functions of downstream miRNAs based on complementary sequences. Mounting studies have revealed close associations between the dysregulation of circRNA-miRNA networks and GC progression (12, 13). For instance, Zhang et al. indicated that circNRIP1 acts as a sponge of miR-149-5p and potentiates the malignant behaviors of GC cells (12). Xie et al. showed that exosomal circSHKBP1 promotes GC malignancy through suppressing the miR-582-3p/HUR/VEGF axis and inhibiting HSP90 degradation (13). Collectively, circRNAs are promising targets for cancer treatment.

Glucose metabolism is the main energy source for cancer growth and development, and is also regarded as a hallmark of cancer. Cancer cells are more dependent on glycolysis, rather than oxidative phosphorylation, to provide sufficient energy (12). Inspired by this phenomenon, researchers have indicated that suppression of cancer glycolysis might be a novel direction for overcoming drug resistance and cancer heterogeneity. Some glycolytic inhibitors, such as genistein, lonidamine and 2-deoxy-D-glucose, have been developed and validated by clinical trials (14–16). However, the existing inhibitors are commonly used as combined drugs due to their limited efficacies and severe adverse reactions in recipients. Some circRNAs, such as circMAT2B, circATP2B1 and circBFAR, have shown capabilities of reprogramming cell glycolysis (9, 17, 18). These evidences suggested that inhibition of important circRNAs may attenuate GC glycolysis and progression. Despite the rapid progress of related basic research, remarkable translational advances in GC therapy are lacking. More circRNA targets should be explored and proved by rigorous validation.

Hsa_circ_0049224 (circDNMT1) is a newly identified circRNA in cancer treatment. It has been reported to be an autophagy modifier and miRNA sponge that potentiates breast cancer development (19, 20). Since the biological functions of circDNMT1 in GC remain unknown, in this study, we aimed to explore the role and underlying mechanisms of circDNMT1 in GC.

## Materials And Methods

### Clinical Specimens

50 pairs of GC and adjacent normal tissues were harvested from patients who were diagnosed with advanced gastric carcinoma at Chinese PLA General Hospital from August 2016 to September 2017. The specimens were stored in liquid nitrogen. These patients did not receive any preoperative chemotherapy or radiotherapy. Informed consent was obtained from the included patients, who were followed-up to determine the 3-year overall survival (OS). This study was approved by the Ethics Committee of Chinese PLA General Hospital.

### Cell Culture

Cell lines were purchased from the Cell Bank of the Chinese Academy of Science (Shanghai, China). HGC-27 cells labeled with firefly luciferase (luc-HGC-27) were previously constructed and stored in our laboratory. Cells were cultivated in Dulbecco’s Modified Eagle’s Medium (DMEM, Thermo Fisher, MA, USA) with supplementary of 10% fetal bovine serum (FBS, Kangyuan, Tianjin, China) and 1% streptomycin/penicillin (Corning, NY, USA). Cells were maintained in a humidified incubator with 5% CO_2_ at 37°C.

### Plasmid Transfection, Lentivirus Packaging and Cell Infection

The small interfering RNAs (siRNAs), short hairpin RNAs (shRNAs), miRNA mimics and overexpression plasmids used in this study were designed and synthesized by JTSBIO Scientific (Wuhan, China, **Table S1**). Lipofectamine 2000 (Thermo Fisher) was used to conduct cell transfection according to the protocol of the manufacture. A Lenti-Pac HIV Expression Packaging Kit (GeneCopoeia, USA) was used to perform the lentivirus packaging. Cells were chemically selected by puromycin (Yuanye, Shanghai, China) at the working solution of 1 μg/mL for 10 days.

### RNA Extraction and Quantitative Real-Time PCR (qRT-PCR)

To detect RNA expression in cells and tissues, RNA extraction and qRT-PCR analysis were performed according to methods described in our previous study (18). The expression of relative genes was analyzed by the 2^-ΔΔCt^ method. Vinculin and U6 served as the internal controls. The oligo dT and qRT-PCR primers are listed in **Table S1**.

### Western Blot (WB) Analysis

Protein was extracted by RIPA buffer (Solarbio, Beijing, China). A BCA Protein Assay Kit (Thermo Fisher) was used to conduct protein quantification of lysed samples. Total protein underwent high-temperature denaturation at 100°C for 15 min. 25 μg of protein was separated by SDS-PAGE and transferred to PVDF membranes (Millipore, MA, USA). The membranes were then incubated with the primary antibodies and the anti-rabbit immunoglobulin G secondary antibody (Abcam, Cambridge, UK). The Super ECL Plus (Biorigin, Beijing, China) was used to make the bands visualized.

### Identification of circDNMT1 Existence

To confirm the authenticity of circDNMT1 detection by qRT-PCR, the amplified product was harvested. Agarose (2%) was prepared with Tris-acetate-EDTA buffer and heated until the agarose was sufficiently dissolved. GelGreen (Biomed, Beijing, China) nucleic acid dye was added into the 2% agarose which was poured into a mold. The mixture solidified into a gel after 20 minutes. Then, the amplified product underwent gel electrophoresis to verify cirDNMT1 existence and observed under ultraviolet irradiation. A 50 bp Ladder DNA Marker (Biomed) was used to show the locations of DNA sizes in the gels. Sanger sequencing was also conducted to compare the nucleotide sequences of the amplified product and circDNMT1 conjunction site.

### RNA Pull-Down Assay

An RNA Pulldown Kit (Geneseed, Guangzhou, China) was used to determine the binding efficiencies between circDNMT1 and miRNAs. The biotin-labeled circDNMT1 probe was mixed with magnetic beads (Thermo Fisher, USA) at room temperature for 2 h. The lysates of HGC-27 and AGS cells were prepared using lysis buffer and sonication, followed by incubation with probes at 4°C for 8 h. The magnetic beads were washed by washing buffer three times. The miRNAs were extracted by Trizol and were analyzed by qRT-PCR. The probe sequences can be viewed in **Table S2**.

### Luciferase Reporter Assay

To examine the binding efficiencies of miR-576-3p, the corresponding wildtype and mutant reporter plasmids as predicted by bioinformatic analysis were constructed (JTSBIO Scientific, Wuhan, China). The miRNA mimics and plasmids were co-transfected into cells. The cells were harvested after 48 h, and the Dual-Luciferase Reporter Assay System (Promega, USA) was used to determine the luciferase activities.

### Fluorescence *In Situ* Hybridization (FISH)

Seeded cells were washed by phosphate buffered saline (PBS) two times and 4% paraformaldehyde was used to fix cells. A Situ Hybridization Kit (BersinBio, Guangzhou, China) and Cy3-labeled circDNMT1 probe (HonyiBio, Guangzhou, China) were used to label circDNMT1 location. The images were recorded using a laser scanning confocal microscope. The circDNMT1 probe sequence can be viewed in **Table S2**.

### Cell Proliferation Assay

For the CCK-8 assay, 3×10^3^ cells were seeded into 96-well plates. After 10 h, the medium was replaced with CCK-8 working solution. The plates were placed at 37° for 1 h protected from light. Then, the absorbance at 450 nm was measured by a microplate reader (Biotech, USA). The experiments were repeated at the indicated times. For the EdU assay, 2×10^4^ cells were cultivated in 96-well plates. The proliferative cells were stained with EdU and 4,6-diamidino-2-phenylindole (DAPI) according to the manufacturers’ protocols. An inverted fluorescence microscope was used to observe the stained cells.

### Cell Migration Assay

To evaluate the capability of GC cells migration, the cells were seeded and the density could reach 100% after 10 h. Cells were vertically scratched using pipette tips and gently washed by PBS for three times. The medium was replaced with DMEM without FBS. The wounds at the specific sites were observed under an inverted microscope at 0 h and 24 h.

### Cell Invasion Assay

The 10% Matrixgel (Corning, NY, USA) was prepared in DMEM. Then, 50 μL of 10% Matrixgel was added to the bottom of the Transwell chamber. 1×10^4^ cells were suspended in 200 μL serum-free DMEM and seeded on a concretionary Matrixgel. The lower chambers were immersed in 600 μL DMEM containing 20% FBS. After 24 h, the cells were stained with 0.1% crystal violet and counted under an inverted microscope.

### Glycolytic Experiments

A Glucose Uptake Assay Kit (Biovision, CA, USA) was used to measure the uptake of glucose. The production of lactate acid, ATP and pyruvic acid was examined by D-Lactate Assay Kit, ATP Colorimetric/Fluorometric Assay Kit and Pyruvate Colorimetric/Fluorometric Assay Kit (Biovision), respectively. The corresponding absorbances were detected using a microplate reader.

### Extracellular Acidification Rate (ECAR) and Oxygen Consumption Rate (OCR) Assays

To further evaluate the metabolism levels of GC cells, ECAR and OCR were determined with a Glycolysis Stress Test Kit and Cell Mito Stress Test Kit (Agilent, USA). Briefly, cells were seeded in Seahorse XF96 plates (Agilent). After 10 h of incubation, the ECAR and OCR were detected according to the manufacture’s protocols. In the detection of ECAR, glucose (10 mM), oligomycin (1 μM) and 2-deoxy-d-glucose (100 mM) were injected into medium of cells sequentially. For the measurement of OCR, cells were treated with oligomycin (1 μM), protonophore trifluoromethoxy carbonyl cyanide phenylhydrazone (FCCP, 0.5 μM) and antimycin A (0.5 μM). The signaling data were read and recorded by a Seahorse XFe96 Analyzer (Agilent).

### Animal Experiments

To establish xenograft models of subcutaneous tumors and lung metastasis, BALB/c nude mice (4 weeks, male, Charles River, Beijing, China) were fed and housed under specific pathogen-free conditions. After 2 weeks of acclimatization, the mice were randomly divided into experimental groups, with 5 mice in each group. For generation of the model with subcutaneous tumors, mice were subcutaneously injected with 1×10^7^ luc-HGC-27 cells suspended in PBS. The tumor volumes were measured by a vernier caliper every 5 days. The evaluation method of tumor volumes as follows: Volume = (length×width^2^)×0.5. 5×10^6^ luc-HGC-27 cells were injected into the tail vein to simulate lung metastasis. After 30 days, 1.5 mg D-Luciferin (Solarbio) was intraperitoneally injected into each mouse. The tumor locations and loads were displayed by bioluminescence. Mice were sacrificed by cervical dislocation after anesthesia.

### Statistical Analysis

The SPSS 25.0 and GraphPad Prism 8.0 software were used to conduct all statistical analysis. The normal distribution was conducted using Q-Q plot and Shapiro-Wilk test. The significance of variables was evaluated by Student’s t-test after distribution examinations. The Chi-square test was used to compare the differences in clinicopathological features. The survival time of included patients was analyzed using Kaplan-Meier method. Data were presented as means ± standard deviation (SD). A p value < 0.05 was considered as the statistical significance.

## Results

### Identification of circDNMT1 Characteristics in GC

**Figure 1:**
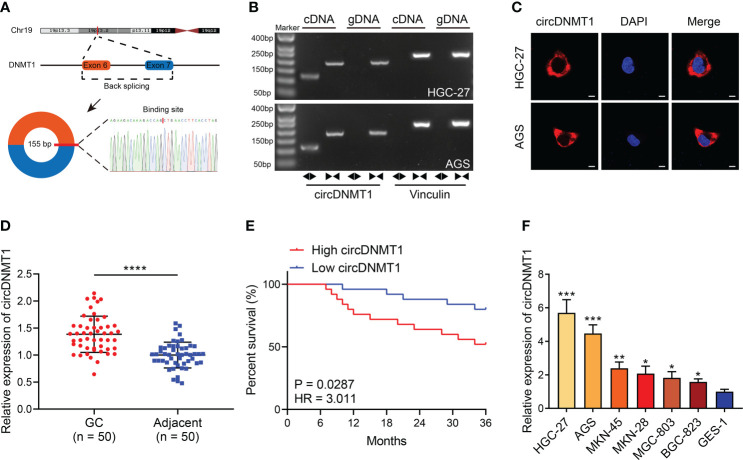
Identification of circDNMT1 characteristics in GC. **(A)** The schematic illustration of circDNMT1 origination and the result of Sanger sequencing. **(B)** The gel electrophoresis to examine amplified product of qRT-PCR using convergent and divergent primers. **(C)** FISH to display the distributions of cricDNMT1 in HGC-27 and AGS cells. Scale bar: 10 μm. **(D)** The qRT-PCR analysis to show circDNMT1 expression in 50 pairs of GC and adjacent normal tissues. **(E)** The Kaplan-Meier plot to show survival time of 50 GC patients who were divided into high-circDNMT1 and low-circDNMT1 groups. **(F)** The qRT-PCR analysis to show circDNMT1 expression in HGC-27, AGS, MKN-45, MKN-28, MGC-803, BGC-823 and GES-1 cell lines. Data were presented as means ± SD. *P < 0.05, **P < 0.01; ***P < 0.001, ****P < 0.0001.

To confirm the existence of circDNMT1 in GC, we first investigated its origins from genomic DNA. circDNMT1 is derived from exon 6 and exon 7 of DNMT1. qRT-PCR primers for sequence amplification across the junction sites were designed. The concrete base sequences of the amplified product were confirmed using Sanger sequencing (**Figure 1A**). The images of DNA gel electrophoresis showed that the circDNMT1 amplification product could only be obtained using divergent primers derived from cDNA. It cannot be amplified with convergent primers or gDNA as templates (**Figure 1B**). FISH images indicated that circDNMT1 was mainly located in the cytoplasm of GC cells (**Figure 1C**). The data identified the existence of circDNMT1 in GC and proved the feasibility of these primers for circDNMT1 amplification.

Next, we measured circDNMT1 expression in clinical specimens from 50 GC patients. circDNMT1 was significantly overexpressed in GC tissues (**Figure 1D**). The median value of circDNMT1 was set as the dividing line between the high-circDNMT1 and low-circDNMT1 groups. The clinicopathological information of these patients was further analyzed. circDNMT1 levels were correlated with pathological T (pT) stage and pathological TNM (pTNM) stage (**Table 1**). High expression of circDNMT1 indicated poorer patient survival time (**Figure 1E**). The circDNMT1 expression in GC and gastric epithelial cell lines was also determined using qRT-PCR analysis. All GC cell lines had higher expression levels than human gastric epithelial cells, and HGC-27 and AGS cells had the highest levels (**Figure 1F**).

**Table 1:** Correlation between circDNMT1 expression and clinicopathological characteristics of 50 GC patients.

Characteristics	Case number	High (n=25)	Low (n=25)	P value
Age at surgery (years)				0.390
<60	29	13	16	
≥60	21	12	9	
Gender				0.208
Male	36	16	20	
Female	14	9	5	
pT stage				**0.037**
pT1+pT2	17	5	12	
pT3+pT4	33	20	13	
Tumor size (cm)				0.157
<5	25	10	15	
≥5	25	15	10	
Location				0.777
Cardiac	23	12	11	
Non-cardiac	27	13	14	
pTNM stage				**0.045**
I+II	21	7	14	
III+IV	29	18	11	
Differentiation				0.157
Poorly	45	24	21	
Well	5	1	4	

The bold values mean the data with significant difference (P < 0.05).

### circDNMT1 Knockdown Inhibits the Proliferation, Migration and Invasion of GC Cells

**Figure 2:**
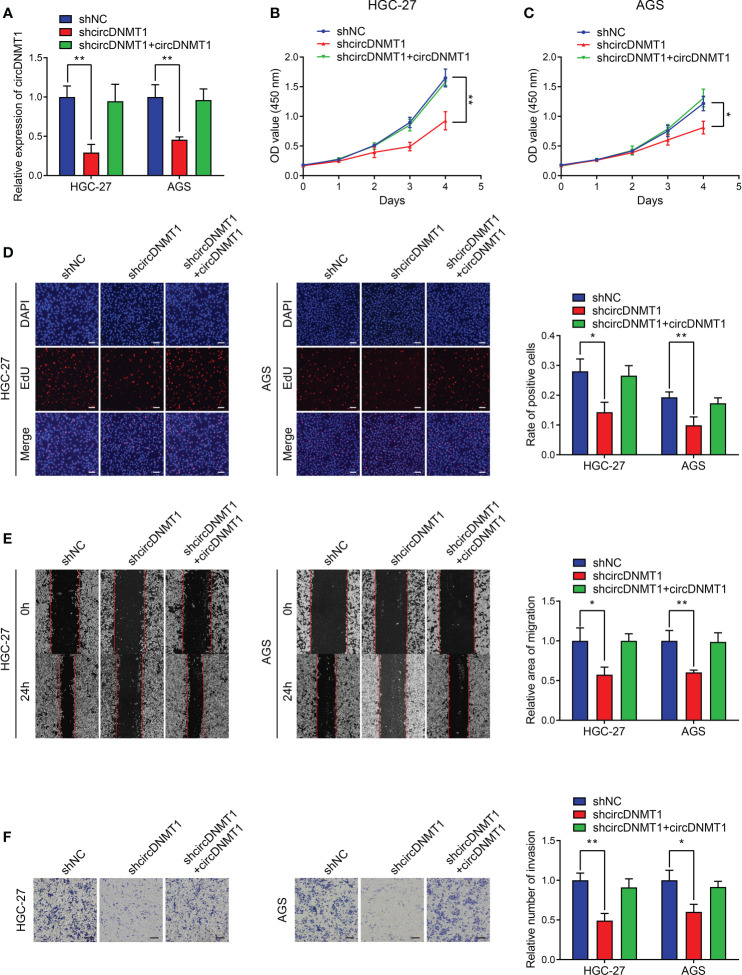
circDNMT1 knockdown inhibits proliferation, migration and invasion of GC cells. **(A)** The qRT-PCR analysis to show circDNMT1 expression in HGC-27 and AGS cells stably carrying lentivirus with NC shRNA or circDNMT1 shRNA and additionally transfected with vector or circDNMT1 overexpression plasmids. **(B, C)** The CCK-8 assay to show proliferation of cells as in **(A)**. **(D)** The EdU assay to show proliferation of cells as in **(A)**. Scale bar: 100 μm. The histogram is displayed on the right. **(E)** The wound healing assay to show migration of cells as in **(A)**. The histogram is displayed on the right. **(F)** The transwell assay to show invasion of cells as in **(A)**. The histogram is displayed on the right. Scale bar: 100 μm. Data were presented as means ± SD. *P < 0.05, **P < 0.01.

Regarding the clinical value of circDNMT1, it has been speculated that circDNMT1 might participate in the progression of GC. The two cell lines with the highest circDNMT1 expression, HGC-27 and AGS, were chosen for the subsequent experiments. We generated the GC cells with stable knockdown of circDNMT1. The interference efficiencies of shRNA and overexpression plasmids were examined using qRT-PCR analysis (**Figures 2A**, **S1**). Downregulation of circDNMT1 expression attenuated the proliferation rates of GC cells, while circDNMT1 overexpression rescued the inhibitory effects on cell proliferation (**Figures 2B** **–D**). circDNMT1 knockdown inhibited GC migration and additional transfection with its overexpression plasmids restored the suppression caused by circDNMT1 knockdown (**Figure 2E**). Transwell assays also verified the effects of circDNMT1 on GC cells invasion (**Figure 2F**). The data of *in-vitro* experiments prove that knockdown of circDNMT1 suppresses the malignant progression of GC cells and there is no off-target effect that interfered with the results.

### circDNMT1 Promotes Glycolysis of GC Cells

**Figure 3:**
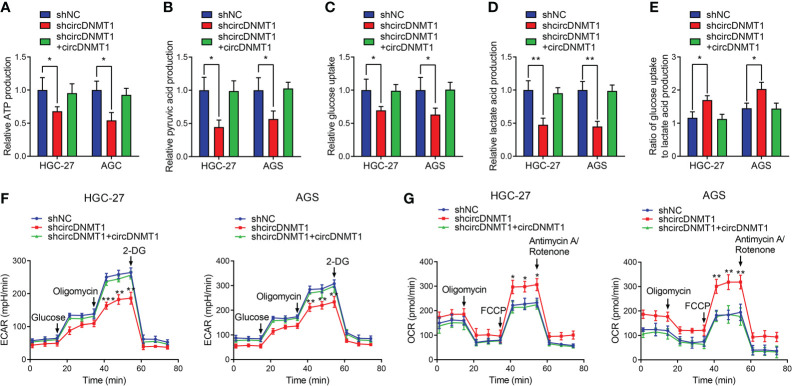
circDNMT1 promotes glycolysis of GC cells. **(A–E)** ATP production **(A)**, pyruvic acid production **(B)**, glucose uptake **(C)**, lactate acid production **(D)** and ratio of glucose uptake to lactate acid production **(E)** were determined in HGC-27 and AGS cells stably carrying lentivirus with NC shRNA or circDNMT1 shRNA and additionally transfected with vector or circDNMT1 overexpression plasmids. **(F, G)** ECAR **(F)** and OCR **(G)** assays of cells as in **(A–E)**. A series of compounds were added at the indicated time. Data were presented as means ± SD. *P < 0.05, **P < 0.01, ***P < 0.001.

Glycolysis is an important initiator of cancer malignancy. To detect glycolysis of GC cells, we determined the metabolite levels using colorimetric methods, which included glucose uptake and lactate acid, ATP, pyruvic acid production. The ratio of glucose uptake to lactate acid production is also an indicator of measuring cell glycolysis. The increased ratio demonstrates the inhibition of glycolysis. Downregulation of circDNMT1 expression suppressed ATP and pyruvic acid production. The decreases of glucose uptake, lactate acid production and a relative increase of their ratio were observed. Restoration of circDNMT1 expression brought them back as the control groups (**Figures 3A** **–E**). The ECAR and OCR represent glycolysis and oxidative phosphorylation, respectively. circDNMT1 knockdown inhibited the ECAR, while the OCR was apparently elevated. This imbalance could be mitigated by additional circDNMT1 overexpression (**Figures 3F, G** **)**. These findings suggest that circDNMT1 may be an important contributor of glycolysis during GC progression.

### miR-576-3p Is Directly Sponged by circDNMT1 in GC

**Figure 4:**
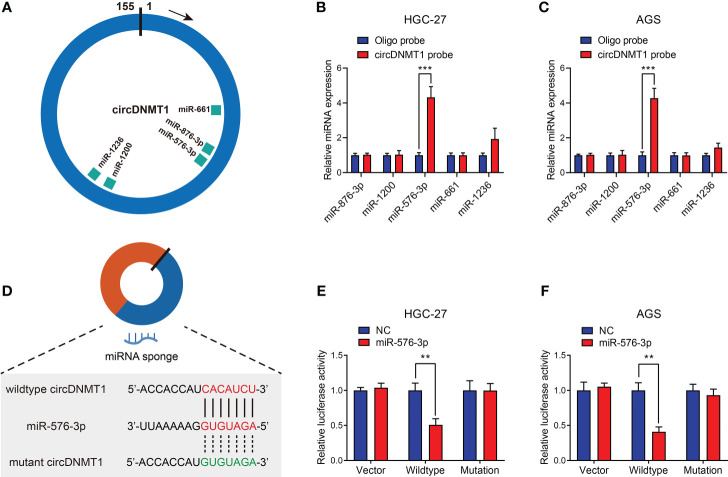
miR-576-3p is directly sponged by circDNMT1 in GC. **(A)** The schematic illustration of miRNAs that can be sponged by circDNMT1 predicted by Circinteractome. **(B, C)** The qRT-PCR analysis to show the miRNA levels that were pulled down by NC probe or circDNMT1 probe in HGC-27 **(B)** and AGS **(C)** cells. **(D)** The schematic illustration of wildtype (red) and mutant (green) sequences of binding sites of circDNMT1 and miR-576-3p. **(E, F)** Luciferase reporter assay to show the relative luciferase activities in HGC-27 **(E)** and AGS **(F)** cells that were cotransfected with empty luciferase reporter plasmids (vector) or plasmids inserted with wildtype or mutant sequences and NC and miR-567-3p mimics. **P < 0.01, ***P < 0.001.

To further reveal the mechanisms of circDNMT1 as a miRNA sponge, we employed a bioinformatic tool, Circinteractome, to predict candidate downstream miRNAs. A total of five miRNAs with over 90 context+ score percentiles were selected out, including miR-876-3p, miR-1200, miR-576-3p, miR-661 and miR-1236 (**Figure 4A**). The probe targeting circDNMT1 was designed and used for baiting miRNAs that directly bind to circDNMT1 in GC cells. Among the five miRNAs, miR-576-3p had the highest binding efficiencies both in HGC-27 and AGS cell lines (**Figures 4B, C** **)**. Next, we constructed the luciferase reporter plasmids that were inserted with wildtype or mutant circDNMT1 sequences (**Figure 4D**). miR-576-3p significantly reduced the luciferase activities of wildtype plasmids in HGC-27 and AGS cells. Whereas, the luciferase activities of mutant plasmids were free from the suppression of miR-576-3p (**Figures 4E, F** **)**. These results suggest that circDNMT1 inhibits miR-576-3p functions by direct complementary binding.

### circDNMT1 Potentiates GC Progression and Glycolysis by Targeting miR-576-3p

**Figure 5:**
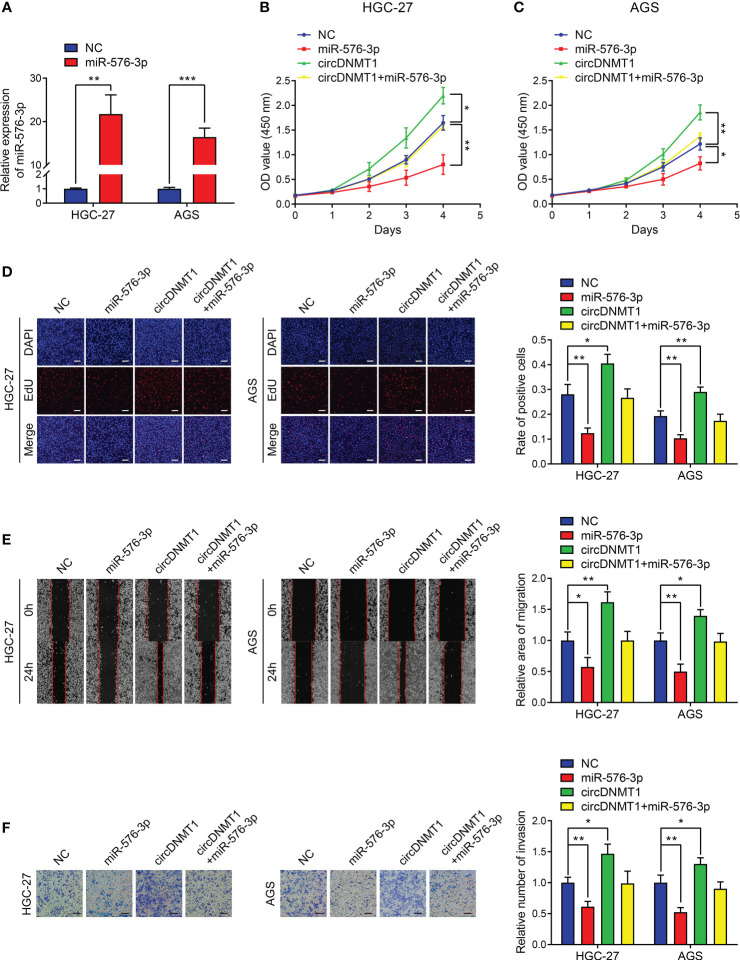
circDNMT1 potentiates GC progression by targeting miR-576-3p. **(A)** qRT-PCR analysis to show the miR-576-3p expression in HGC-27 and AGS cells that were transfected with NC or miR-576-3p mimics. **(B, C)** The CCK-8 assay to show proliferation of HGC-27 **(B)** and AGS **(C)** cells stably carrying lentivirus with vectors or circDNMT1 overexpression plasmids and additionally transfected with NC or miR-576-3p mimics. **(D)** The EdU assay to show proliferation of cells as in (B-C). Scale bar: 100 μm. The histogram is displayed on the right. **(E)** The wound healing assay to show migration of cells as in **(B, C)**. The histogram is displayed on the right. **(F)** The transwell assay to show invasion of cells as in **(B, C)**. The histogram is displayed on the right. Scale bar: 100 μm. Data were presented as means ± SD. *P < 0.05, **P < 0.01, ***P < 0.001.

The oncological functions of miR-576-3p in GC were not reported. Thus, we examined the effects of miR-576-3p on the malignant phenotypes of GC cells. We designed miR-576-3p mimics, and their overexpression efficiencies were examined by qRT-PCR (**Figure 5A**). The *in-vitro* experiments showed that upregulation of miR-576-3p suppressed GC proliferation, migration and invasion. circDNMT1 overexpression counteracted the inhibition induced by miR-576-3p (**Figures 5B** **–F**). For cell glycolysis, miR-576-3p had inhibitory effects on glucose uptake, lactate acid, ATP, pyruvic acid production and promotive effects on the ratio of glucose uptake to lactate acid production (**Figures S2A–E**). The ECAR was attenuated while the OCR was inversely enhanced. The data show that circDNMT1 could mitigate the effects of miR-576-3p (**Figures S2F, G**).

### miR-576-3p Suppresses Hypoxia Inducible Factor-1 Alpha (HIF-1α) Expression by Directly Binding to Its mRNA 3’UTR

miRNAs can inhibit downstream gene expression by binding its mRNA 3’UTR and accelerating mRNA degradation. miR-576-3p has been reported to regulate HIF-1α expression in cancer (21, 22). Therefore, it was speculated that circDNMT1 could promote GC progression by sponging miR-576-3p/HIF-1α axis. HIF-1α was inhibited by miR-576-3p while circDNMT1 overexpression elevated its expression. circDNMT1 could also mitigate the inhibitory effects of miR-576-3p (**Figure S3A**). We then aimed to confirm the mechanistic associations between miR-576-3p and HIF-1α. According to the sites predicted by Targetscan, we designed luciferase reporter plasmids that were inserted with wildtype or mutant sequences of the mRNA 3’UTR of HIF-1α (**Figure S3B**). miR-576-3p could suppress the activities of wildtype luciferase reporter plasmids instead of mutant ones (**Figures S3C, D**). These data prove that miR-576-3p directly suppresses HIF-1α expression in GC cells.

### circDNMT1 Promotes GC Malignancy by Targeting the miR-576-3p/HIF-1α Axis

**Figure 6:**
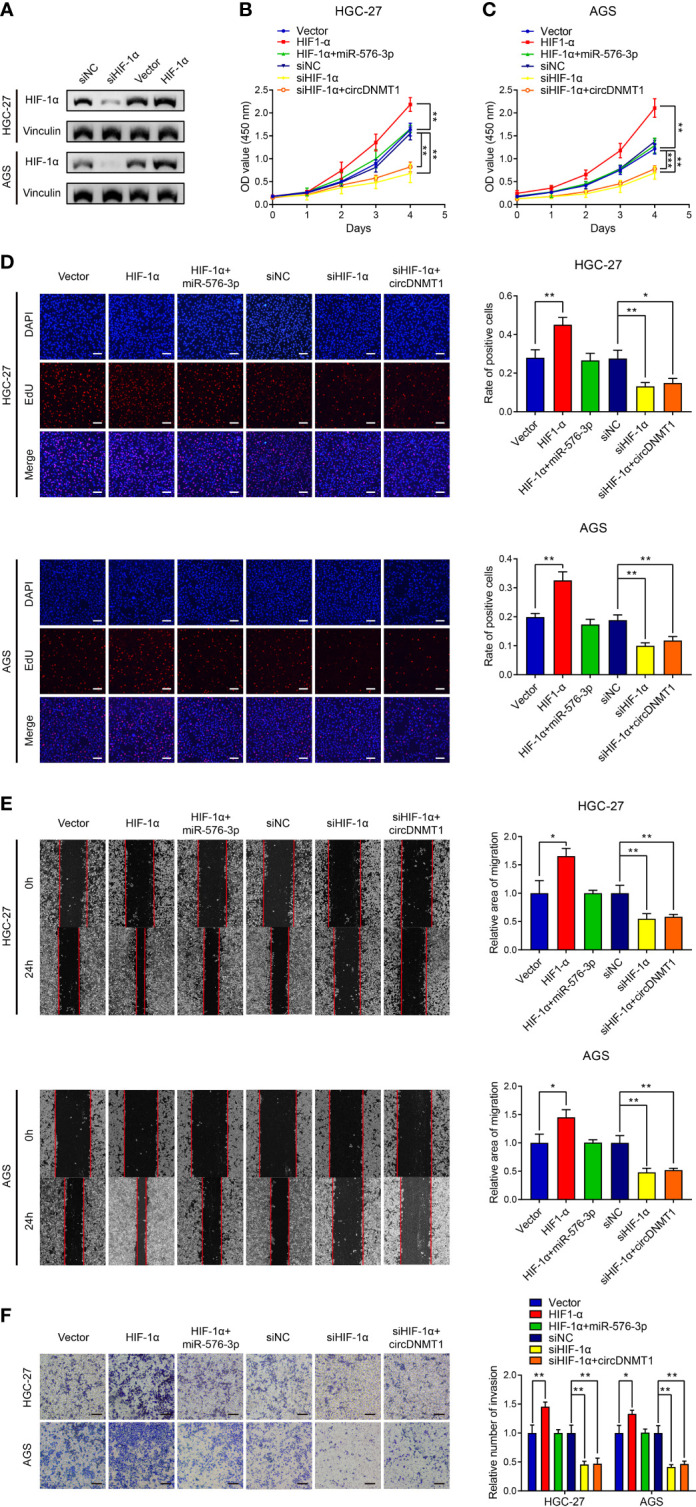
circDNMT1 promotes GC malignancy by targeting miR-576-3p/HIF-1α axis. **(A)** The WB analysis to show HIF-1α expression in HGC-27 and AGS cells transfected with NC shRNA or HIF-1α siRNA, or carrying lentivirus with vector or HIF-1α overexpression plasmids. **(B, C)** The CCK-8 assay to show proliferation of HGC-27 **(B)** and AGS **(C)** cells. They could be divided into two groups: i) The cells stably carrying lentivirus with vector or HIF-1α overexpression plasmids and transfected with NC or miR-576-3p mimics; ii) The cells transfected with NC shRNA or HIF-1α siRNA and carrying lentivirus with vector or circDNMT1 overexpression plasmids. **(D)** The EdU assay to show proliferation of cells as in **(B, C)**. Scale bar: 100 μm. The histogram is displayed on the right. **(E)** The wound healing assay to show migration of cells as in **(B, C)**. The histogram is displayed on the right. **(F)** The transwell assay to show invasion of cells as in **(B, C)**. The histogram is displayed on the right. Scale bar: 100 μm. Data were presented as means ± SD. *P < 0.05, **P < 0.01, ***P < 0.001.

We then knocked down HIF-1α using a specific siRNA, and the results were verified by WB analysis (**Figure 6A**). HIF-1α overexpression potentiated the proliferation of GC cells, and miR-576-3p could rescue the effects of HIF-1α overexpression. HIF-1α knockdown significantly reduced the proliferation capability of GC cells. However, this proliferative inhibition could not be effectively rescued by circDNMT1 overexpression (**Figures 6B** **–D**). Cell migration and invasion had the similar tendencies as proliferation detection. miR-576-3p and HIF-1α had the counteracting effects. Elimination of HIF-1α functions abrogated promotion induced by circDNMT1 (**Figures 6E, F** **)**. These findings show that circDNMT1 potentiates GC malignant behaviors by targeting the miR-576-3p/HIF-1α axis. HIF-1α may serve as a mediator of circDNMT1 to contribute to GC progression.

### The circDNMT1/miR-576-3p/HIF-1α Axis Regulates GC Growth and Metastasis *In Vivo*

**Figure 7:**
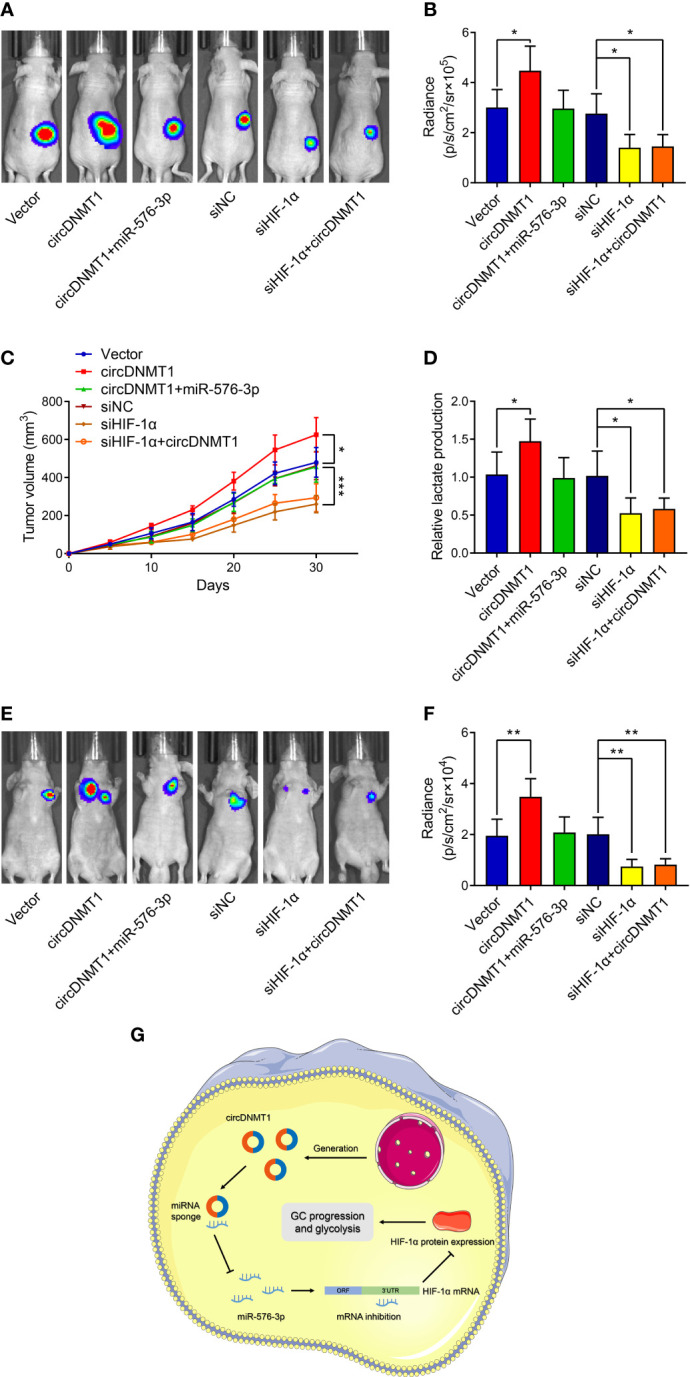
circDNMT1/miR-576-3p/HIF-1α axis regulated GC growth and metastasis *in vivo*. **(A)** Representative bioluminescence images at 30 days after subcutaneous injection of luc-HGC-27 cells. They could be divided into two groups: i) The cells stably carrying lentivirus with vector or HIF-1α overexpression plasmids and transfected with NC or miR-576-3p mimics; ii) The cells transfected with NC shRNA or HIF-1α siRNA and carrying lentivirus with vector or circDNMT1 overexpression plasmids. **(B)** Luminescence signals in **(A)** represented by overlaid false-color images with the signal intensity. **(C)** Curves of tumor volumes as in **(A)** at the indicated time. **(D)** Lactate acid production was determined in the tumor tissues from **(A)**. **(E)** Representative bioluminescence images at 30 days after tail vein injection of luc-HGC-27 cells as in **(A)**. **(F)** Luminescence signals in **(E)** represented by overlaid false-color images with the signal intensity. **(G)** Schematic illustration of the role and mechanisms of circDNMT1 in regulating GC progression. Data were presented as means ± SD. *P < 0.05, **P < 0.01, ***P < 0.001.

To further verify the participation of the circDNMT1/miR-576-3p/HIF-1α axis in GC development, generation of mouse models with subcutaneous tumor and lung metastasis was conducted. circDNMT1 overexpression facilitated the *in-vivo* growth of GC. Transfection of miR-576-3p mimics into tumors mitigated the promotive effects. Consistent with the *in-vitro* experiments, HIF-1α knockdown induced decreases in cancer growth and abrogated the functions of circDNMT1 (**Figures 7A** **–C**). The lactate acid concentrations in cell-derived tumors were determined, which were in line with the tendencies of tumor growth (**Figure 7D**). Moreover, upregulation of circDNMT1 expression potentiated GC metastasis, which was counteracted by miR-576-3p. Downregulation of HIF-1α expression impaired the metastasis, and circDNMT1 could not rescue these inhibitory effects (**Figures 7E, F** **)**. Collectively, our work identified the oncogenic role of circDNMT1 in GC and found that the miR-576-3p/HIF-1α axis might serve as the main downstream pathway of it as shown by both *in-vitro* and *in-vivo* experiments (**Figure 7G**).

## Discussion

The research concerning biological functions of circRNA is a hot topic in recent years. circRNA participate in the regulation of many diseases, such as cardiovascular diseases, neural degradation, and endocrine disorders (23–25). Numerous studies have elucidated the mechanisms of circRNA regulatory network during the processes of carcinogenesis and metastasis. circRNA-5692 attenuates the malignant behaviors of hepatocellular carcinoma by suppressing the miR-328-5p/DAB2IP axis (26). circNDUFB2 reduces the stabilization of IGF2BPs and enhances anti-tumor immunity in lung cancer (27). The mechanistic associations between circRNAs and GC have also been studied. Dysregulation of circRNA expression serves as a hallmark of GC development and has potential value of diagnosis and prognostic prediction (28). Interference with expression of some circRNAs could reverse GC progression and mitigate tumor burdens both *in vitro* and *in vivo* (12, 13). However, few clinical trials about circRNAs in GC treatment have been conducted. It may be attributable to the limited findings of existing reports. Therefore, it is eagerly required to explore more effective targets and to broaden horizons of the circRNA landscape in cancer development.

circDNMT1 is a novel circRNA that has been reported to promote breast cancer progression. circDNMT1 was overexpressed in breast cancer tissues. As an miRNA sponge, circDNMT1 maintains the highly proliferative state of breast cancer by regulating the miR-485-3p/ZEB1 axis (20). Another study showed that circDNMT1 promotes nuclear translocation of p53 and AUF1 and activates autophagy to increase survival capabilities of breast cancer (19). Our findings showed that circDNMT1 exhibited overexpression in GC. The clinicopathological features and 3-year OS of GC patients were collected and circDNMT1 expression indicated clinical severity and poor survival. circDNMT1 indicated shorter survival time of GC patients. Further experiments demonstrated that circDNMT1 knockdown led to inhibition of GC progression. Inversely, its upregulation could promote these malignant behaviors. circDNMT1 may have potentials as a valuable target in clinical practice of GC.

Aerobic glycolysis was firstly identified by Otto Warburg in the last century. Glycolysis can provide adequate energy and substance supply to fulfill the active state of many types of cancer cells. This metabolic reprogramming links to various malignant phenotypes, including rapid proliferation, chemoresistance formation and immune evasion (17, 29, 30). Enlightened by this evidence, researchers began to investigate the value of glycolysis inhibition in cancer therapy. As important regulators of cancer progression, some circRNAs have also been proved to affect glycolysis to modulate GC malignancy (9, 17, 18). We next speculated that circDNMT1 might facilitate cancer development by reprogramming glucose metabolisms. Downregulation of circDNMT1 expression attenuated glycolytic indicators. The ECAR reflects general glycolysis levels, while the OCR serves as an index of oxidative phosphorylation. Knockdown of circDNMT1 reduced the ECAR whereas enhanced the OCR. It suggested that GC cells rely more on oxidative phosphorylation to provide energy after the suppression of aerobic glycolysis. The data proved that circDNMT1 is a critical regulator of GC glycolysis both *in vitro* and *in vivo*, which might be a contributor of cancer development.

The role of circRNAs as miRNA sponges has received more attention from researchers compared to other circRNA mechanisms. The circRNA-miRNA regulatory network has been depicted by many studies (12, 13). Regarding the oncogenic role of circDNMT1, we aimed to investigate the downstream mechanisms of its functions. Combinational use of bioinformatic analysis and further experiments indicated high binding efficiencies between circDNMT1 and miR-576-3p based on the complementary sequences. There are significantly counteractive effects of circDNMT1 and miR-576-3p on GC malignant behaviors. Zuo et al. reported that miR-576-3p overexpression sensitizes ovarian cancer to cisplatin by reducing PD−L1 and cyclin D1 expression (31). It also mitigates the migration and invasion capabilities of lung adenocarcinoma (32). The researches about the functions of miR-576-3p in GC were absent. Our findings reported the tumor suppressive role of miR-576-3p in GC, which is under rigorous regulation of circRNA networks.

HIF-1α is a classical transcription factor that regulates the expression of many oncogenic factors and tumor suppressors. HIF-1α activity is also under the control of complicated regulatory networks in cancer cells (33, 34). HIF-1α promotes GC growth and metastasis by regulating miR-17-5p/PDCD4 axis (35). It also facilitate angiogenesis by upregulating VEGF-A expression and macrophage polarization (36). As a cell sensor of microenvironment, HIF-1α modulates glycolytic activity to sustain cancer viability and progression (37, 38). To further clarify the downstream mechanisms of circDNMT1, we consulted the existing studies concerning miR-576-3p functions in cancer. Two studies reported that HIF-1α expression is suppressed by miR-576-3p at the post-transcriptional level in glioma and hepatocellular carcinoma (21, 22). In addition, HIF-1α overexpression contributes to glycolysis promotion of cancer (39). Therefore, we speculated that circDNMT1 potentiated GC progression by sponging miR-576-3p/HIF-1α axis. A library of interaction experiments confirmed high binding efficiencies between the HIF-1α mRNA 3’UTR and miR-576-3p. Overexpression of HIF-1α mitigated its inhibitory effects on GC malignant behaviors. Moreover, the loss of circDNMT1/miR-576-3p function was observed after HIF-1α knockdown. These findings demonstrated that HIF-1α is a main target of the circDNMT1/miR-576-3p axis.

There are some limitations of our work. First, glycolysis is an important mediator of cancer development. In the present study, we only verified that circDNMT1 simultaneously enhanced GC malignancy and glycolysis. Its functions in glycolysis-mediated progression should be confirmed by further experiments. Second, HIF-1α might serve as a critical downstream of circDNMT1, which does not mean HIF-1α is the only target. More research should be performed to reveal the circRNA-miRNA networks in GC. Third, whether this axis regulates malignancy in other types of cancer remains to be explored.

Collectively, this study identified the oncogenic role of circDNMT1 in GC. circDNMT1 was overexpressed in GC and was correlated with the clinicopathological characteristics and poor prognosis of GC patients. It promoted GC proliferation, migration, invasion and glycolysis by targeting the 576-3p/HIF-1α axis both *in vitro* and *in vivo*. circDNMT1 might become a prognostic factor and therapeutic target in clinical practice of GC treatment.

## Data Availability Statement

The original contributions presented in the study are included in the article/**Supplementary Material**. Further inquiries can be directed to the corresponding author.

## Ethics Statement

The studies involving human participants were reviewed and approved by Ethical Committee of Chinese PLA General Hospital. The patients/participants provided their written informed consent to participate in this study. The animal study was reviewed and approved by Ethical Committee of Animal Center of Chinese PLA General Hospital.

## Author Contributions

BW designed the study and supervised the process of research. HL, BC, and RZ performed the experiments, collected data, and wrote the original draft. TL, XX, HC, HD, and JG analyzed and visualized the data. All authors contributed to this research and approved the final version.

## Funding

This study was funded by National Natural Science Foundation of China (No. 82073192 and 81773135).

## Conflict of Interest

The authors declare that the research was conducted in the absence of any commercial or financial relationships that could be construed as a potential conflict of interest.

## Publisher’s Note

All claims expressed in this article are solely those of the authors and do not necessarily represent those of their affiliated organizations, or those of the publisher, the editors and the reviewers. Any product that may be evaluated in this article, or claim that may be made by its manufacturer, is not guaranteed or endorsed by the publisher.
