# Supplementary material for: circDNMT1 Promotes Malignant Progression of Gastric Cancer Through Targeting miR-576-3p/Hypoxia Inducible Factor-1 Alpha Axis
Source: Front Oncol. 2022 May 30;12:817192. doi: 10.3389/fonc.2022.817192 (PMC9197105; doi:10.3389/fonc.2022.817192)
Supplement: Supplementary file 1 [file DataSheet_1.docx]

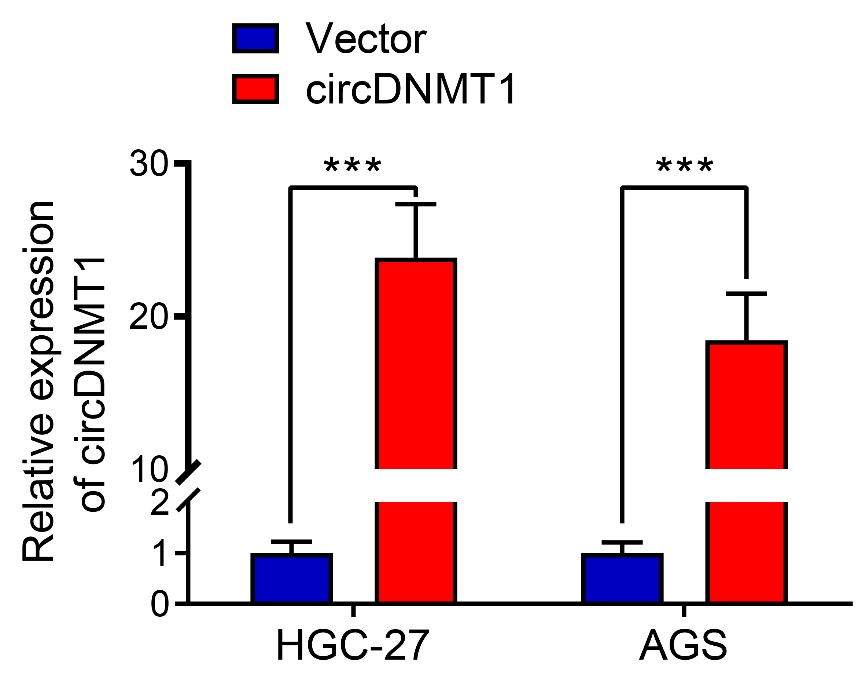


**Supplementary Figure 1.** Confirmation of efficiencies of circDNMT1 expression plasmids. qRT-PCR analysis to show circDNMT1 in HGC-27 and AGS cells stably carrying lentivirus with vectors or circDNMT1 overexpression plasmids. Data were presented as means±SD. ***P < 0.001.
