# Supplementary material for: circDNMT1 Promotes Malignant Progression of Gastric Cancer Through Targeting miR-576-3p/Hypoxia Inducible Factor-1 Alpha Axis
Source: Front Oncol. 2022 May 30;12:817192. doi: 10.3389/fonc.2022.817192 (PMC9197105; doi:10.3389/fonc.2022.817192)
Supplement: Supplementary file 2 [file DataSheet_2.docx]

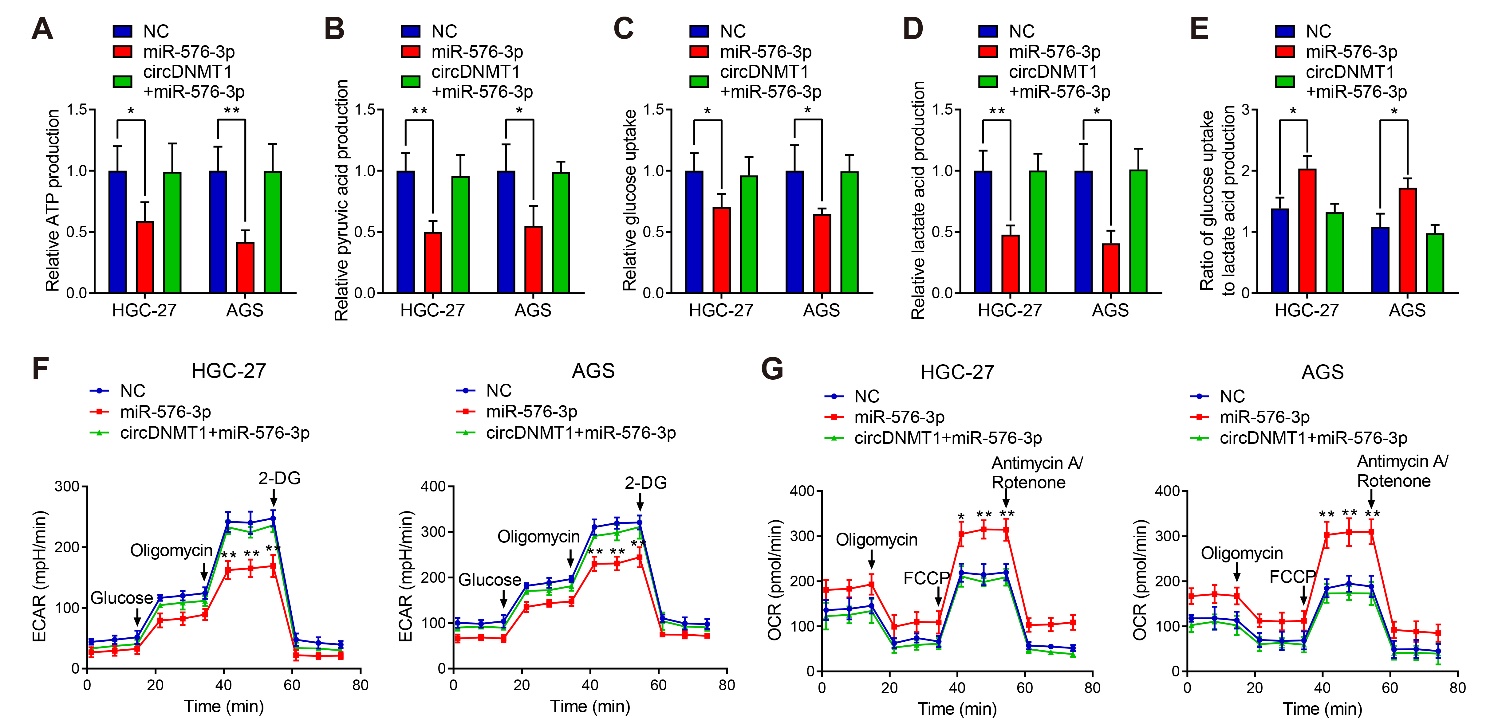


**Supplementary Figure 2.** circDNMT1/miR-576-3p axis regulates GC glycolysis. circDNMT1 promotes glycolysis of GC cells. (A-E) ATP production (A), pyruvic acid production (B), glucose uptake (C), lactate acid production (D) and ratio of glucose uptake to lactate acid production (E) were determined in HGC-27 and AGS cells stably carrying lentivirus with NC shRNA or circDNMT1 shRNA and additionally transfected with vector or circDNMT1 overexpression plasmids. (F, G) ECAR (F) and OCR (G) assays of cells as in (A-E). A series of compounds were added at the indicated time. Data were presented as means±SD. *P < 0.05, **P < 0.01.
