# Supplementary material for: circDNMT1 Promotes Malignant Progression of Gastric Cancer Through Targeting miR-576-3p/Hypoxia Inducible Factor-1 Alpha Axis
Source: Front Oncol. 2022 May 30;12:817192. doi: 10.3389/fonc.2022.817192 (PMC9197105; doi:10.3389/fonc.2022.817192)
Supplement: Supplementary file 3 [file DataSheet_3.docx]

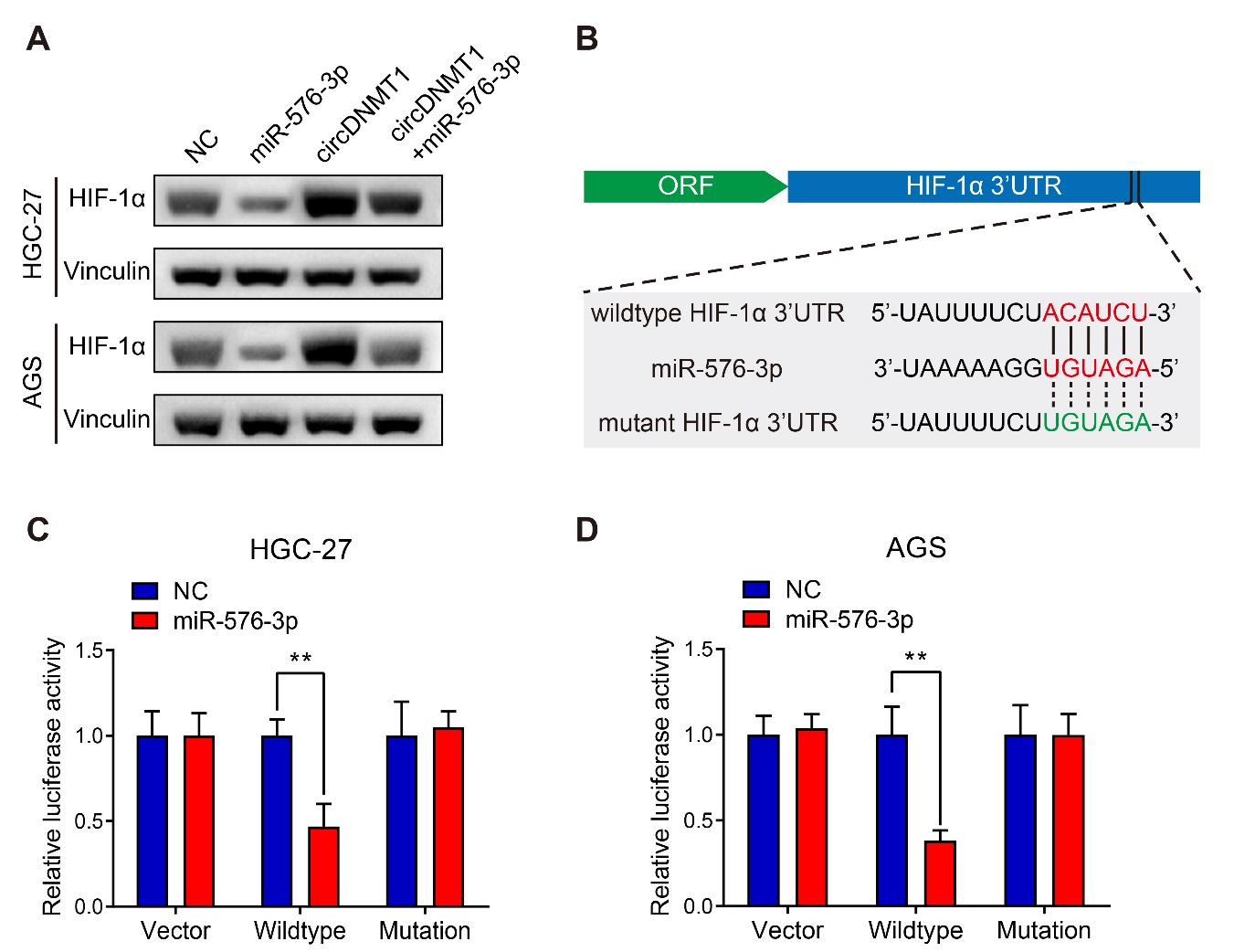


**Supplementary Figure 3.** miR-576-3p suppresses HIF-1α expression by directly binding to its mRNA 3’UTR. (A) WB analysis to show the expression of HIF-1α in HGC-27 and AGS cells stably carrying lentivirus with vectors or circDNMT1 overexpression plasmids and additionally transfected with NC or miR-576-3p mimics. Vinculin served as the internal control. (B) The schematic illustration of wildtype (red) and mutant (green) sequences of binding sites of 3’UTR of HIF-1α mRNA and miR-576-3p. (C, D) Luciferase reporter assay to show the relative luciferase activities in HGC-27 (C) and AGS (D) cells that were cotransfected with empty luciferase reporter plasmids (vector) or plasmids inserted with wildtype or mutant sequences and NC and miR-567-3p mimics. **P < 0.01.
