# Supplementary material for: circDNMT1 Promotes Malignant Progression of Gastric Cancer Through Targeting miR-576-3p/Hypoxia Inducible Factor-1 Alpha Axis
Source: Front Oncol. 2022 May 30;12:817192. doi: 10.3389/fonc.2022.817192 (PMC9197105; doi:10.3389/fonc.2022.817192)
Supplement: Supplementary file 4 [file Table_1.docx]

**Table S1 The sequences of primers and oligo dT for qRT-PCR used in this study.**

| Target | Primer | Sequence (5’-3’) |
| --- | --- | --- |
| circDNMT1 | Forward primer | GAAAGAGCCAAATCGGATGA |
|  | Reverse primer | GCCCTTTGCAAAATGAGATG |
| DNMT1 | Forward primer | CAGCACAAACTGACCTGCTT |
|  | Reverse primer | AGCCAGTGATCCACCATTCA |
| miR-876-3p | oligo dT | GTCGTATCCAGTGCAGGGTCCGAGGTATTCGCACTGGATACGACTGAATT |
|  | Forward primer | TGGTGGTTTACAAAGT |
| miR-1200 | oligo dT | GTCGTATCCAGTGCAGGGTCCGAGGTATTCGCACTGGATACGACGAGGCT |
|  | Forward primer | CTCCTGAGCCATTCTG |
| miR-576-3p | oligo dT | GTCGTATCCAGTGCAGGGTCCGAGGTATTCGCACTGGATACGACGATTCC |
|  | Forward primer | AAGATGTGGAAAAATT |
| miR-661 | oligo dT | GTCGTATCCAGTGCAGGGTCCGAGGTATTCGCACTGGATACGACACGCGC |
|  | Forward primer | TGCCTGGGTCTCTGGCCT |
| miR-1236 | oligo dT | GTCGTATCCAGTGCAGGGTCCGAGGTATTCGCACTGGATACGACTCCCCA |
|  | Forward primer | TGAGTGACAGGGGAAA |
| Universal primer for miRNAs | | GTGCAGGGTCCGAGGT |
| Vinculin | Forward primer | CCAAGATGATTGACGAGAGACAG |
|  | Reverse primer | AGAGGTGAGTTGTAACACACGA |
| U6 | Forward primer | CTCGCTTCGGCAGCACA |
|  | Reverse primer | AACGCTTCACGAATTTGCGT |
