# Supplementary material for: circDNMT1 Promotes Malignant Progression of Gastric Cancer Through Targeting miR-576-3p/Hypoxia Inducible Factor-1 Alpha Axis
Source: Front Oncol. 2022 May 30;12:817192. doi: 10.3389/fonc.2022.817192 (PMC9197105; doi:10.3389/fonc.2022.817192)
Supplement: Supplementary file 5 [file Table_2.docx]

**Table S2 The sequences of oligonucleotides and probes used in this study**

| Definition | Sequence (5’-3’) |
| --- | --- |
| shNC | Sense: TTCTCCGAACGTGTCACGT |
|  | Antisense: ACGTGACACGTTCGGAGAA |
| shcircDNMT1 | Sense: GACAAAGACCAGCTGAACCTT |
|  | Antisense: AAGGTTCAGCTGGTCTTTGTC |
| siNC | Sense: TTCTCCGAACGTGTCACGTTT |
|  | Antisense: AAACGTGACACGTTCGGAGAA |
| siHIF-1α | Sense: CGATGGAAGCACTAGACAAAG |
|  | Antisense: TTGTCTAGTGCTTCCATCGGA |
| miR-576-3p mimics NC | Sense: UUCUCCGAACGUGUCACGU |
|  | Antisense: ACGUGACACGUUCGGAGAA |
| miR-576-3p mimics | Sense: AAGAUGUGGAAAAAUUGGAAUC |
|  | Antisense: GAUUCCAAUUUUUCCACAUCUU |
| Oligo probe | TTTGCTTGTCTTTTCCCTGA |
| Biotin-circDNMT1 probe | GACCAGCTGAACCTTCACCTA |
| Cy3-labeled circDNMT1 probe | GACCAGCTGAACCTTCACCTA |
